# Research on the Influence of Orthogonal Design Optimized Elicitor Combinations on Fucoxanthin Accumulation in *Phaeodactylum tricornutum* and Its Expression Regulation

**DOI:** 10.3390/md23060244

**Published:** 2025-06-09

**Authors:** Han Yang, Yifu Gong, Boyue Liu, Yuru Chen, Huan Qin, Heyu Wang, Hao Liu

**Affiliations:** 1 Key Laboratory of Marine Biotechnology of Zhejiang Province, School of Marine Sciences, Ningbo University, Ningbo 315200, China; qingkui_yanghan@163.com (H.Y.); lby2025@foxmail.com (B.L.); 15168152325@163.com (Y.C.); ruxu_qin.nb@foxmail.com (H.Q.); hao_liu@biotranstech.com (H.L.); 2 School of Food Sciences and Engineering, Ningbo University, Ningbo 315832, China; wangheyu@nbu.edu.cn; 3 Institute of Bioengineering, Biotrans Technology Co., Ltd., Shanghai 201500, China; 4 United New Drug Research and Development Center, Biotrans Technology Co., Ltd., Changsha 410000, China

**Keywords:** *Phaeodactylum tricornutum*, fucoxanthin, orthogonal experiment, photosynthetic physiological index, gene expression

## Abstract

Fucoxanthin, a carotenoid with notable pharmaceutical potential, has attracted significant attention due to its efficient accumulation in marine microalgae and the importance of optimizing its induction conditions. In this study, *Phaeodactylum tricornutum* was employed as a model organism to screen optimal conditions for fucoxanthin accumulation using a three-factor, four-level orthogonal design. Furthermore, the underlying mechanisms related to photosynthetic physiology and gene regulation were explored. The results revealed that both glycine (Gly) and light intensity significantly enhanced fucoxanthin content (*p* < 0.05). The optimal condition (Combination C: 0.50 g L^−1^ Gly, 36 μmol photons·m^−2^·s^−1^, 12 h light/12 h dark) yielded a fucoxanthin content of 0.87 μg g^−1^, representing a 35% increase compared to the control. Meanwhile, Combination *p* (0.50 g L^−1^ Gly, 36 μmol photons·m^−2^·s^−1^, 24 h light/0 h dark) significantly improved cell density (5.11 × 10^6^ cells mL^−1^; +18%) and fucoxanthin yield (4.10 μg L^−1^; +47%). Analysis of photosynthetic parameters demonstrated that the non-photochemical quenching coefficient (NPQ) was suppressed. Gene expression profiling showed that Combination C upregulated photosynthetic genes (*psbA*, *rbcL*, *rbcS*) by up to 2.36-fold, while Combination P notably upregulated *fcpb* (7.59-fold), *crtiso*, and *pds*. Principal component analysis identified that *rbcS* and *pds* are key regulatory genes. These findings demonstrate that Gly, light intensity, and photoperiod synergistically regulate the expression of genes involved in photosynthesis and carotenoid biosynthesis, thereby promoting fucoxanthin accumulation. This work provides valuable insights and a theoretical basis for optimizing fucoxanthin production in support of marine drug development.

## 1. Introduction

*Phaeodactylum tricornutum*, a member of the diatom family, holds a pivotal role in marine ecosystems and various scientific research domains. Compared to large brown macroalgae such as *Saccharina japonica* and *Sargassum*, *P. tricornutum* exhibits superior photosynthetic efficiency, rapid growth rates, and notably higher fucoxanthin content [1] As such, it is recognized as an ideal and sustainable algal resource for fucoxanthin production [2]. Fucoxanthin, a carotenoid compound [3], is a marine-derived bioactive substance with beneficial biological activities, including anti-inflammatory [4], antioxidant [5], anti-obesity [6], and anti-cancer effects [7]. Moreover, fucoxanthin contributes to lowering blood glucose and lipid levels [8], reducing the risk of cardiovascular diseases and modulating immune functions [9], and it exhibits synergistic effects when combined with chemotherapeutic agents [10]. However, the natural fucoxanthin content in *P. tricornutum* is exceptionally low, accounting for only approximately 1% of its dry weight [11]. Consequently, the high cost of fucoxanthin-based pharmaceuticals poses significant barriers to industrial-scale production. Therefore, developing cost-effective strategies to enhance fucoxanthin accumulation remains an urgent challenge that demands resolution.

Appropriate light conditions are beneficial for the accumulation of secondary metabolites in microalgae. However, prolonged exposure to continuous illumination triggers photoprotective mechanisms in algal cells, leading to the degradation of fucoxanthin and subsequently suppressing its biosynthesis to prevent photodamage [12]. Thus, optimizing both light intensity and photoperiod is crucial for enhancing fucoxanthin synthesis. It has been reported that, compared to a lower light intensity of 54 μmol photons·m^−2^·s^−1^, an elevated intensity of 1000 μmol photons·m^−2^·s^−1^ resulted in reductions of 47.63%, 29.19%, and 16.23% in biomass, intracellular protein, and polysaccharide content, respectively, in native microalgae [13]. With increasing light intensity, the relative growth rate and weight gain of *Branchioglossum farlowianum* significantly improved, whereas the contents of chlorophyll a (Chl a), carotenoids, phycoerythrin, and phycocyanin generally exhibited a declining trend [14]. While the effects of light intensity vary across algal species, excessively high light intensity is generally detrimental to algal growth and metabolite accumulation. Similarly, different algal species respond differently to photoperiods. In *Isochrysis zhanjiangensis*, fucoxanthin content peaked under a 6 h light/18 h dark cycle, while maximum fucoxanthin yield was achieved under a 12 h light/12 h dark cycle [15]. For *Cylindrotheca closterium*, fucoxanthin accumulation reached its highest level under an 18 h light/6 h dark cycle [16]. Appropriate light intensity and photoperiod thus promote both growth and secondary metabolite accumulation in algal cells. Glycine (Gly), as an organic nitrogen source, is widely used to study its effects on the growth and development of plants and algae. Gly has been shown to enhance the rapid growth of *Synechocystis sauvageau* and *Chlorella vulgaris*, stimulate the accumulation of primary metabolites such as sugars, organic acids, and amino acids, and increase levels of plant hormones, fructose, total soluble sugars, and glycogen [17] Additionally, Gly promotes both the content and yield of triptolide in *Tripterygium wilfordii* Hook. f. [18]. While Gly, light intensity, and photoperiod have been individually reported to promote algal growth, no prior study has explored their combined effects on *P. tricornutum* and its fucoxanthin accumulation.

Orthogonal experiment is an efficient method for investigating multi-factor, multi-level experimental systems. Prior studies have demonstrated that the optimal combination of 25 mg L^−1^ phenylalanine, 15 mg L^−1^ arginine, 20 mg L^−1^ proline, and 15 mg L^−1^ Gly effectively promotes the growth of *Dunaliella salina* [19], while the combination of 1 μg L^−1^ photosynthetic induction factor (PIF), 0.20 mg L^−1^ ammonium cerium sulfate (ACS), and 40 mg L^−1^ acetylsalicylic acid (ASA) enhances fucoxanthin accumulation in *P. tricornutum* [20]. These findings underscore the efficiency of the orthogonal experiment, which allows for the elucidation of multi-factorial interactions with a minimal number of experimental runs. Optimal light intensity is beneficial for increasing the biomass of *Polygonatum sibiricum* seedlings, with chlorophyll content peaking at 360 μmol photons·m^−2^·s^−1^ in line 83 of this species [21]. Each plant species has its preferred light intensity, under which the yield of secondary metabolites is maximized [22]. Full light exposure promotes the biosynthesis and accumulation of luteoloside in *Lonicera japonica Thunb.*, and under 100% light intensity, the expression levels of light-responsive transcription factors involved in flavonoid metabolism—such as NAC, WD40, MYB, ERF, and WRKY—are markedly upregulated [23]. Photoperiod can also influence the chlorophyll content in the leaves of *Yulania denudata var. purpurascens*, where both excessively long and short light durations adversely affect chlorophyll synthesis. A light-dark cycle of 14 h light/10 h dark is most favorable for its growth, and photoperiod has been shown to significantly affect chlorophyll fluorescence parameters [24]. Additionally, an appropriate photoperiod promotes the accumulation of secondary metabolites in plants [22], while under long-day conditions, the expression of multiple genes involved in flavonoid biosynthesis pathways in *Ipomoea batatas*—including *CHI*, *F3H*, *DFR*, and *UFGT*—is significantly upregulated [25]. Glycine, as an organic nitrogen source, is widely utilized in plant research. At appropriate concentrations, Gly has been reported to enhance photosynthetic pigment content in various plant species [26,27], facilitate photosynthesis in *Phaeocystis globosa* at its optimal concentration [28], and promote secondary metabolism, as evidenced by the peak accumulation of triptolide in *T. wilfordii* at 1 mg L^−1^ Gly concentration [18]. To date, however, no studies have reported on the orthogonal effects of Gly, photoperiod, and light intensity on fucoxanthin accumulation in *P. tricornutum*. Whether the orthogonal combination of these three factors could synergistically affect the growth, fucoxanthin content and yield, Chl a content, chlorophyll fluorescence parameters, and the expression of key photosynthetic and fucoxanthin biosynthesis genes in *P. tricornutum* remains unclear. Therefore, this study employs an orthogonal design incorporating three factors at four levels to systematically explore the combined effects of Gly, light intensity, and photoperiod on *P. tricornutum*. By comprehensively investigating their impacts on photosynthesis and secondary metabolism and elucidating the underlying molecular mechanisms, this study aims to provide robust theoretical and practical foundations for the accumulation of marine-derived bioactive compounds and the further advancement of industrial-scale applications.

## 2. Results

### 2.1. Effects of Orthogonal Experimental Design on Fucoxanthin Accumulation in P. tricornutum

An orthogonal experimental approach was employed to investigate the effects of different combinations of exogenous inducers on the fucoxanthin content and yield in *P. tricornutum*. The results (Table 1, Table 2 and Table 3) demonstrated that the optimal inducer combination for enhancing fucoxanthin content was 0.50 g L^−1^ Gly + 36 μmol photons·m^−2^·s^−1^ + 12 h light/12 h dark (Combination C), while the optimal combination for promoting both cell growth and fucoxanthin yield was0.50 g L^−1^ Gly + 36 μmol photons·m^−2^·s^−1^ + 24 h light/0 h dark (Combination P). Data analysis revealed that both Gly and light intensity had extremely significant effects on fucoxanthin content and yield in *P. tricornutum*, whereas photoperiod had a significant effect on fucoxanthin content but no significant effect on fucoxanthin yield. According to the F-values, the order of importance of the three factors influencing fucoxanthin accumulation was: light intensity > Gly > photoperiod. Validation experiments confirmed these findings, showing that under Combination C, the fucoxanthin content reached 0.87 μg g^−1^, representing a 35% increase compared to the control group (CK). Under Combination P, the cell density reached 5.11 × 10^6^ cells mL^−1^, an 18% increase over the CK group, while the fucoxanthin yield reached 4.10 μg L^−1^, which was 47% higher than the CK group.

### 2.2. Effects of the Optimal Inducer Combinations on Cell Density of P. tricornutum

**Figure 1:**
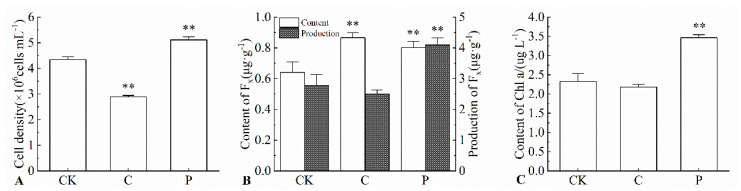
Effects of Combination C and P on physiological indicators of *P. tricornutum.* (**A**) shows the cell density under different treatment conditions. Continuous light (Combination P) significantly promoted biomass accumulation, with the highest density reaching 5.11 × 10^6^ cells mL^−1^ (an 18% increase compared to the CK), while the 12-h light-dark cycle (Combination C) exhibited lower growth density (2.89 × 10^6^ cells mL^−1^). (**B**) illustrates fucoxanthin content and yield under different treatments. The fucoxanthin content under Combination C reached 0.87 μg g^−1^ (a 35% increase compared to the CK). Notably, the fucoxanthin yield of Combination P (4.10 μg L^−1^) was achieved through the multiplicative effect of its high cell density (5.11 × 10^6^ cells mL^−1^) and moderate content (0.80 μg g^−1^), representing a 47% increase over the CK and significantly superior to the yield of Combination C (2.52 μg L^−1^). (**C**) shows the Chl a content under different treatments. The content under Combination P was significantly enhanced (3.46 g L^−1^, a 49% increase), while Combination C showed a slight decrease compared to the control group. “**” indicates that, compared with the CK group, there is a highly significant statistical difference in the corresponding indicators (cell density, fucoxanthin content/yield, Chl a content) in this group, with a corresponding *p*-value less than 0.01 (*p* < 0.01).

The effects of Combination C and *p* on the cell density of *P. tricornutum* were evaluated. The results (Figure 1.) showed that the cell densities under Combination C and P were 2.89 × 10^6^ cells mL^−1^ and 5.11 × 10^6^ cells mL^−1^, respectively, corresponding to 0.66-fold and 1.18-fold of the CK group. These findings indicate that, compared with the CK group, Combination C suppressed algal cell growth, while Combination P promoted algal cell proliferation.

### 2.3. Effects of the Optimal Inducer Combinations on Fucoxanthin Content and Yield in P. tricornutum

The effects of Combination C and P on fucoxanthin content and yield in *P. tricornutum* were examined. As shown in Figure 1, the fucoxanthin content and yield under Combination C were 0.87 ng g^−1^ and 2.50 ng g^−1^, representing 1.35-fold and 0.90-fold of the CK group, respectively. Under Combination P, fucoxanthin content and yield reached ng g^−1^ and 4.10 ng g^−1^, which correspond to 1.25-fold and 1.47-fold of the CK group, respectively. Compared with the CK group, Combination C promoted the accumulation of fucoxanthin content. However, due to the extremely significantly lower cell density in Combination C than in the CK group, the yield of Combination C decreased. Combination P promoted fucoxanthin content, and the cell density was extremely significantly higher than that of the CK group. Under the dual effects of fucoxanthin content and cell density, the yield of Combination P was extremely significantly higher than that of the CK group.

### 2.4. Effects of the Optimal Inducer Combinations on Chl a Content in P. tricornutum

The impacts of Combination C and P on the Chl a content in *P. tricornutum* were investigated. The results (Figure 1) indicated that the Chl a content under Combination C and P was 2.18 g L^−1^ and 3.46 g L^−1^, respectively, equivalent to 0.94-fold and 1.49-fold of the CK group. This indicates that, compared with CK, Combination C inhibited the accumulation of Chl a in *P. tricornutum*, while Combination P promoted an increase in Chl a content.

### 2.5. Effects of the Optimal Inducer Combinations on Chlorophyll Fluorescence Parameters in P. tricornutum

**Figure 2:**
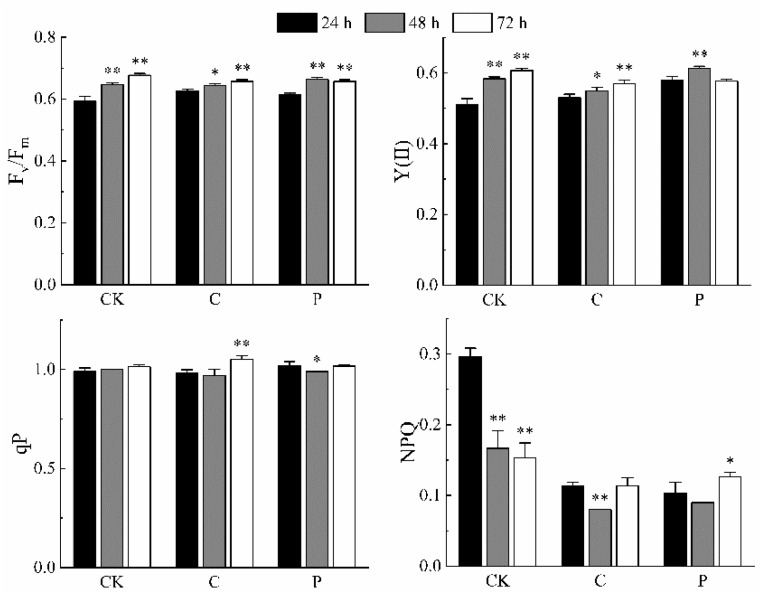
Chlorophyll fluorescence parameters of *P. tricornutum* under the optimal inducer combination conditions. The figures above show the maximum photochemical quantum yield of PS II (F_v_/F_m_), the actual photochemical quantum yield of PS II [Y(II)], the photochemical quenching coefficient (qP), and the non-photochemical quenching coefficient (NPQ), respectively. Under the three different culture combination conditions, the four chlorophyll fluorescence parameters all changed to varying degrees, among which the change in NPQ was the most significant. “*” indicates that, compared with the CK group, there is a significant statistical difference in the corresponding indicators (Fv/Fm, Y (II), qP, NPQ) for this group, with a corresponding *p*-value less than 0.05 (*p* < 0.05). This shows that such a difference is unlikely to be caused by random error and has statistical significance. “**” represents that, in comparison with the CK group, there is a highly significant statistical difference in the corresponding indicators for this group, with a corresponding *p*-value less than 0.01 (*p* < 0.01). The probability of the difference being caused by random factors is lower, and the result has higher reliability.

The effects of Combination C and P on the chlorophyll fluorescence parameters of *P. tricornutum* were assessed. The results (Figure 2) showed that with prolonged treatment time, both the maximum quantum yield of PSII (Fv/Fm) and effective quantum yield of PSII [Y(II)] exhibited significant to highly significant increases. Under Combination C, the photochemical quenching coefficient (qP) showed a highly significant increase on the third day, whereas under Combination P, qP significantly decreased on the second day. Moreover, NPQ values under both Combination C and P were significantly lower than those of the CK group.

### 2.6. Effects of the Optimal Inducer Combinations on the Expression of Fucoxanthin-Related Genes in P. tricornutum

**Figure 3:**
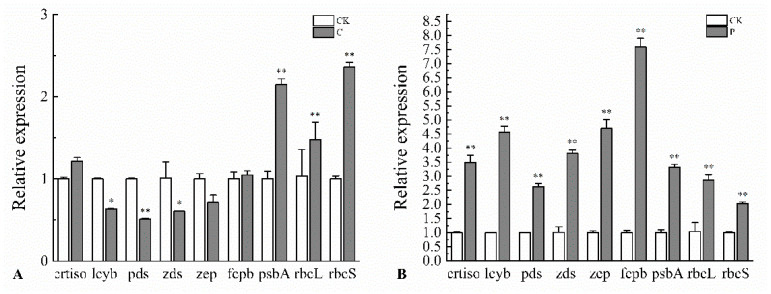
Effects of Combination C (**A**) and P (**B**) on the transcription of various genes in *P. tricornutum*. (**A**) shows the relative expression levels of key enzyme genes in the fucoxanthin synthesis pathway (crtiso, lcyb, pds, zds, zep) and key photosynthetic enzyme genes (fcpb, psbA, rbcL, rbcS) under the culture conditions of Combination C, and their comparison with those of CK. Similarly, (**B**) shows the relative expression levels of the above nine genes under the culture conditions of Combination P and their comparison with those of CK. “*” indicates that the relative expression level of the corresponding gene in this group (dark-colored bars) shows a significant statistical difference compared with the CK group (white bars), with a corresponding *p*-value less than 0.05 (*p* < 0.05). This suggests that the difference is unlikely to be caused by random error and is statistically signifi-cant. “**” indicates that the relative expression level of the corresponding gene in this group exhibits a highly significant statistical difference compared with the CK group, with a corresponding *p*-value less than 0.01 (*p* < 0.01). This indicates a lower probability that the difference is due to random factors, and the result is more reliable.

The effects of the optimal inducer combinations on the expression of key photosynthetic enzyme genes and fucoxanthin biosynthetic pathway genes in *P. tricornutum* were investigated. The results (Figure 3) showed that, compared with the CK group, Combination C significantly upregulated the expression of photosynthetic enzyme genes *psbA*, *rbcL* and *rbcS*, with *rbcS* exhibiting the highest expression level at 2.36 times that of the CK group. Under Combination P, the expression levels of photosynthetic enzyme genes *psbA*, *rbcL*, *rbcS* and *fcpb*, as well as fucoxanthin biosynthetic pathway genes *pds*, *lcyb*, *zds*, *crtiso* and *zep*, were all significantly upregulated, with *fcpb* showing the highest expression at 7.59 times that of the CK group. These findings indicate that the combined effects of Gly, light intensity, and photoperiod enhance fucoxanthin accumulation in *P. tricornutum*, which is closely associated with the upregulation of key genes involved in photosynthesis and the fucoxanthin biosynthetic pathway.

### 2.7. Correlation and Principal Component Analysis of Fucoxanthin with Related Parameters in P. tricornutum

The correlations between fucoxanthin content and yield with Chl a content, chlorophyll fluorescence parameters, photosynthesis-related genes (*crtiso*, *lcyb*, *pds*, *zds*, *zep*), and key fucoxanthin biosynthetic pathway genes (*fcpb*, *psbA*, *rbcL*, *rbcS*) were analyzed. The results (Table 4) indicated that fucoxanthin content was significantly positively correlated with Fv/Fm and Y(II), highly significantly positively correlated with qP and *rbcS* gene expression, and highly significantly negatively correlated with NPQ. Fucoxanthin yield showed highly significant positive correlations with Chl a content and the expression levels of *crtiso*, *lcyb*, *pds*, *zds*, *zep,* and *fcpb* genes, as well as significant positive correlations with Y(II) and the expression of *psbA* and *rbcL* genes.

**Figure 4:**
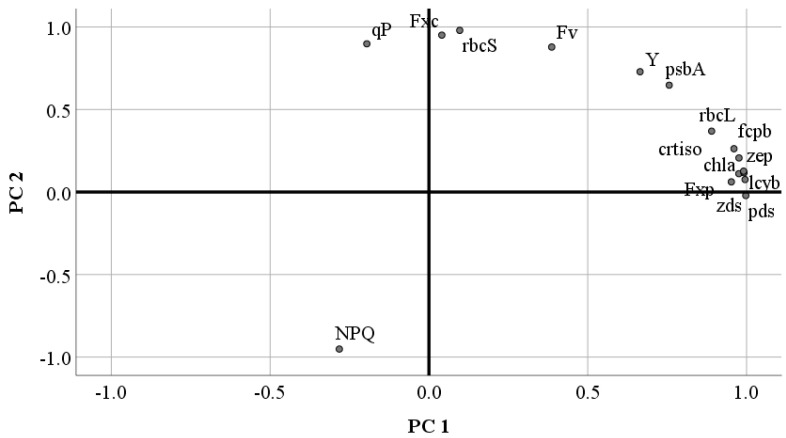
Principal component analysis of *P. tricornutum*.

The results of the principal component analysis (Figure 4) showed that all parameters were reduced to two principal components (PC1 and PC2). PC1 was primarily associated with the *pds* gene, contributing 61.05% of the total variance, while PC2 was primarily associated with the *rbcS* gene, bringing the cumulative contribution to 96.02%. These findings indicate that *pds* and *rbcS* are the principal factors influencing fucoxanthin accumulation.

Principal component analysis is a dimensionality-reduction statistical method used to transform multiple correlated variables into a few uncorrelated comprehensive variables (i.e., principal components) so as to more clearly display the relationships and differences among data. Indicators that are close to each other have a high degree of similarity in the comprehensive characteristics represented by the principal components and may be related in terms of physiological functions or regulatory mechanisms. Indicators distributed in different quadrants indicate that they are related to different comprehensive variables in principal component analysis and may play different roles in the physiological processes of *P. tricornutum*. This figure helps to screen out the key indicators that have a significant impact on the physiological state of *P. tricornutum* or processes such as fucoxanthin synthesis. It can also be used to discover potential synergistic or antagonistic relationships among different indicators, providing directions for further research.

## 3. Discussion

Orthogonal experimental design is an efficient approach that strategically arranges factors and levels to obtain comprehensive information with a relatively small number of experimental runs. Its advantages include balanced dispersion and clear comparability, allowing for the exploration of interactions among multiple factors, which often promotes the accumulation of secondary metabolites. Research shows that an optimal mix of 0.02 g L^−1^ Na_2_CO_3_, initial pH 7, *n*/P ratio of 30, 5% inoculum size, 25 °C and 158.4 μmol photons·m^−2^·s^−1^ significantly accelerates *Chlorella* sp.’s growth rate [29]. When 400 μmol L^−1^ fosfomycin and 50 μmol L^−1^ lovastatin are applied and the sampling time is set at 6 days, which are the optimal conditions for inhibiting the accumulation of secondary metabolites in *Gentiana macrophylla*, according to research, the contents of loganic acid, swertiamarin, gentiopicroside, and sweroside are decreased by 69%, 36%, 33%, and 4%, respectively [30]. These studies demonstrate that orthogonal experimental designs are both feasible and efficient for regulating algal growth and secondary metabolite accumulation. In our study, the combination of 0.50 g L^−1^ Gly + 36 μmol photons·m^−2^·s^−1^ + 12 h light/12 h dark significantly enhanced fucoxanthin content in *P. tricornutum* by 35% compared with the CK group, while the combination of 0.50 g L^−1^ Gly + 36 μmol photons·m^−2^·s^−1^ + 24 h light/0 h dark significantly increased both cell density and fucoxanthin yield by 18% and 47%, respectively, over CK. These findings are consistent with previous research.

The Chl a content in Combination P was significantly higher than that in groups CK and Combination C, while the difference in Chl a content between groups CK and Combination C was relatively small. Chlorophyll a is an important photosynthetic pigment in photosynthesis, participating in the absorption, transfer, and conversion of light energy. The increase in Chl a content in Combination P indicates that under its treatment conditions, the synthesis of chlorophyll a may have been promoted. More chlorophyll a can absorb more light energy, providing a greater energy basis for photosynthesis, which is conducive to improving photosynthetic efficiency and photosynthetic yield. Chlorophyll fluorescence parameters directly reflect the functional state of PSII and are highly sensitive indicators of physiological status and growth potential in plants [31]. In this study, the Fv/Fm values of the CK, Combination C and P groups on the second and third days were significantly higher than those on the first day, indicating an improvement in the maximum potential photochemical efficiency of PSII under dark-adapted conditions. The Y(II) value in Combination P initially increased but subsequently declined, likely due to algal cells adapting to the Gly concentration and light intensity in the early stages; however, prolonged continuous illumination (24 h light/0 h dark) and high light intensity may have led to PSII reaction center closure or damage, causing photoinhibition. This phenomenon explains the reduction in fucoxanthin content under Combination P. During photoinhibition, the rate of reactive oxygen species (ROS) production exceeds the scavenging capacity of antioxidant enzymes, leading to the degradation of the D1 protein. When PSII centers are subjected to excessive light energy, structural changes in the D1 protein make it more susceptible to proteolytic cleavage. Algal cells possess inherent self-repair and protective mechanisms. Once the D1 protein is damaged, cells initiate a turnover process in which newly synthesized D1 proteins are transported to PSII to replace the impaired ones [32]. This process explains the increased expression of *psbA*. In Combination C, qP exhibited a highly significant increase on the third day, indicating a greater degree of PSII center openness, more effective conversion of light energy into chemical energy, enhanced electron transport activity, and improved photosynthetic efficiency. Under these conditions, microalgae can utilize more light energy within the limited photoperiod, accounting for the higher fucoxanthin content observed in Combination C, though the low cell density resulted in lower overall fucoxanthin yield. The NPQ values of Combination C and P at different time points showed significant changes and were reduced to varying degrees relative to group CK at each time point. The NPQ of group CK was extremely significantly higher than those of Combination C and P. This may be due to changes in the photosynthetic-metabolic resource re-allocation mechanism and the thylakoid lumen acidification response. In the short term, the decrease in NPQ was accompanied by a significant increase in the expression of psbA and rbcS genes, which may promote the allocation of light energy to the Calvin cycle by reducing non-radiative energy dissipation. This is consistent with the trend of the increase in fucoxanthin content. However, under continuous strong light treatment, the continuous low-level NPQ accompanied by fluctuations in Y(II) may imply a decline in the ability to maintain the proton gradient, which is consistent with the NPQ-Fv/Fm uncoupling phenomenon in the Phyllostachys edulis drought experiment. This indicates that under the conditions of group CK, the absorbed light energy increased while the photosynthetic capacity decreased, forcing the plant to protect itself by increasing heat dissipation. Therefore, the fucoxanthin content, fucoxanthin yield, and Chl a content in group CK were low.

**Figure 5:**
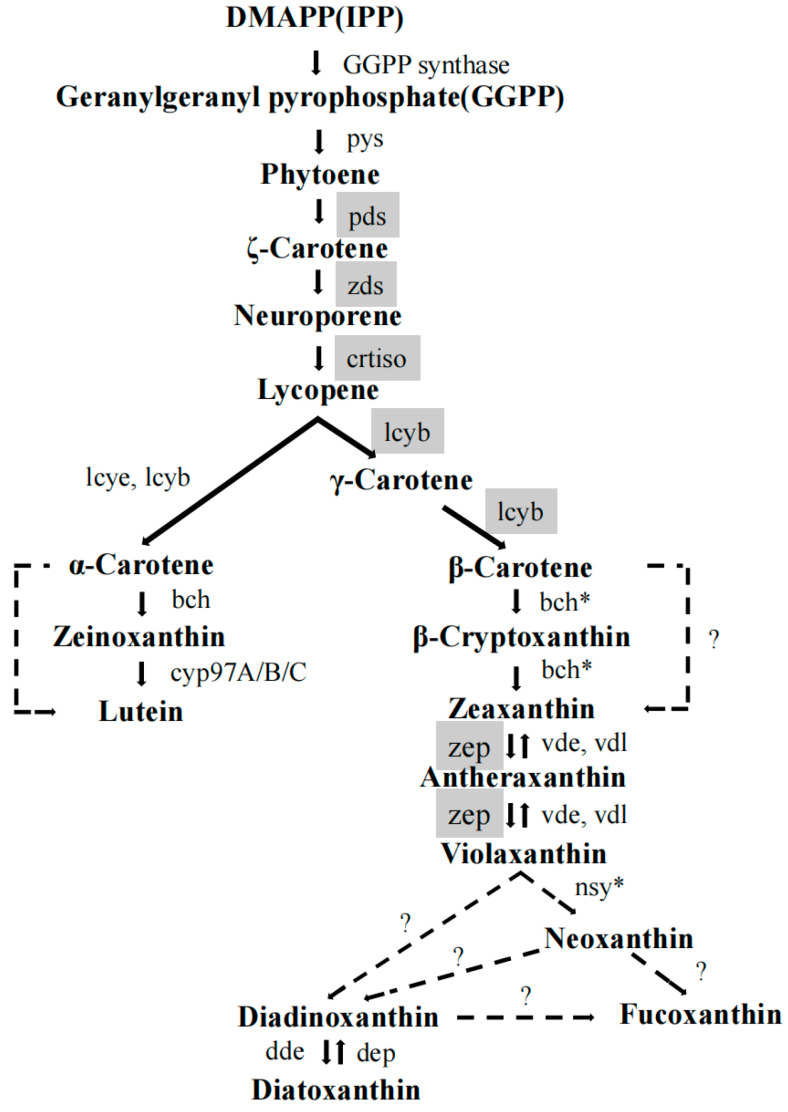
Schematic diagram of fucoxanthin biosynthetic pathway in algae. (Redrawn based on the literature by Mikami [34] and Liang [35]) Carotenoid biosynthesis begins with the head-to-tail linkage of two C20 geranylgeranyl pyrophosphate (GGPP) molecules, which are catalyzed by the *pys* synthase to form phytoene. The synthesis process of lycopene and its associated enzymes are relatively conserved across all algae. Through the catalytic action of a series of enzymes, fucoxanthin is ultimately synthesized. The “?” in the diagram indicates an unknown enzyme in this synthetic pathway, signifying that the identity of this enzyme remains uncharacterized in algae. "*" indicates that the enzyme is unknown in algae. The five key enzymes involved in fucoxanthin biosynthesis selected in this experiment are highlighted in gray in the figure.

Figure 5 shows the synthesis pathway of fucoxanthin in *P. tricornutum*. The *rbcS* and *pds* genes are critically important in the growth of *P. tricornutum* and the accumulation of fucoxanthin. The *rbcS* gene encodes the small subunit of ribulose-1,5-bisphosphate carboxylase/oxygenase (Rubisco), which, together with the large subunit encoded by *rbcL*, constitutes Rubisco [33]. As the key enzyme in the Calvin cycle, Rubisco catalyzes the fixation of CO_2_ by binding it with ribulose-1,5-bisphosphate to produce two molecules of 3-phosphoglycerate, thereby converting inorganic carbon into organic carbon. This process provides carbon skeletons for subsequent metabolic activities and biosynthesis, playing a pivotal role in determining photosynthetic efficiency and carbon assimilation capacity. Given that *rbcS* participates in the core steps of carbon fixation, its expression level directly affects the photosynthetic carbon assimilation efficiency in *P. tricornutum*, influencing algal growth and biomass accumulation. In this study, *rbcS* expression levels under both Combination C and P were significantly higher than those in the CK group, indicating enhanced Rubisco activity that facilitated more efficient CO_2_ fixation and supplied more carbon skeletons for cellular growth and division, ultimately increasing biomass. A sufficient carbon supply is fundamental for fucoxanthin biosynthesis, as carbon assimilation regulated by *rbcS* provides necessary precursors. The CO_2_ fixed during photosynthesis is metabolized into various intermediates that participate in the fucoxanthin biosynthetic pathway, supplying essential substrates for fucoxanthin synthesis. Therefore, *rbcS* indirectly regulates fucoxanthin biosynthesis through its role in enhancing carbon assimilation, thereby contributing significantly to increased fucoxanthin production.

The *pds* gene encodes phytoene desaturase, a key enzyme in the fucoxanthin biosynthetic pathway. *Pds* catalyzes the conversion of phytoene to ζ-carotene [36], a critical step in carotenoid biosynthesis. Subsequently, ζ-carotene undergoes further enzymatic reactions to produce intermediates such as lycopene, eventually leading to the formation of fucoxanthin. Positioned upstream in the carotenoid biosynthetic pathway, variations in the activity and expression of *pds* can influence the overall metabolic flux of this pathway. Elevated *pds* expression enhances the flow of precursors toward carotenoid synthesis, thereby increasing the availability of substrates for fucoxanthin production. Moreover, *pds* acts synergistically with other enzymes involved in carotenoid biosynthesis, forming a complex enzymatic network. Changes in *pds* activity can affect the balance and efficiency of this network, thereby impacting the rate and yield of fucoxanthin biosynthesis.

## 4. Materials and Methods

### 4.1. Cultivation of P. tricornutum

The algal strain of *P. tricornutum* was provided by the Microalgae Laboratory of Ningbo University. The culture was maintained in MVA medium [37] and incubated in a top-illuminated LED growth chamber at 20 °C, with the algal suspension thoroughly agitated at regular intervals each day.

### 4.2. Orthogonal Experimental Design

Gly, light intensity and photoperiod were selected as the key experimental factors. Based on these, an L12(3^4^) orthogonal array was employed to construct an experimental framework consisting of three factors at four levels (Table 5). The specific levels of these three factors were selected based on preliminary experiments conducted prior to the commencement of this study. Details of the orthogonal array and the twelve treatment combinations derived from it are comprehensively listed in Table 2. Each treatment was conducted in triplicate.

### 4.3. Determination of P. tricornutum Cell Density

When *P. tricornutum* cultures reached the stationary phase, cell density in each group was determined following the method described by Rongshi Chen [38].

### 4.4. Determination of Fucoxanthin Content and Yield in P. tricornutum

Upon reaching the stationary phase, the fucoxanthin content and yield in each group of *P. tricornutum* were measured according to the method of Yiheng Song [15]. Among them, the fucoxanthin content refers to the amount of fucoxanthin in a single algal cell, while the fucoxanthin yield is the product of the number of algal cells and the fucoxanthin content in a single cell, i.e., the total amount of fucoxanthin in all algal cells.

### 4.5. Data Analysis of Orthogonal Experimental Results

SPSS 26.0 software was used to perform descriptive statistics and analysis of variance (ANOVA) on the data from the 16 orthogonal test groups to evaluate the effects of Gly, light intensity and photoperiod on the growth of *P. tricornutum* as well as fucoxanthin content and yield. Based on the mean values of cell density, fucoxanthin content and yield under each factor, the optimal levels of Gly concentration, light intensity and photoperiod were determined. These were then combined according to the criteria of highest cell density, fucoxanthin content and yield to derive the three optimal inducer combinations for *P. tricornutum*. We named the elicitor combination with the highest fucoxanthin content Combination C. Experimental results showed that the elicitor combination with the highest cell density of *P. tricornutum* was consistent with that with the highest yield, so we uniformly named it Combination P.

### 4.6. Validation of Orthogonal Experimental Results

Based on the three optimal inducer combinations that promoted the highest cell density, fucoxanthin content and yield, *P. tricornutum* was recultivated. Upon reaching the stationary phase, cell density, fucoxanthin content and yield were measured in each group, and SPSS was used for variance analysis.

### 4.7. Determination of Chl a Content, Fucoxanthin Content and Yield Under the Optimal Inducer Combination

When cultures reached the stationary phase during the validation experiments, cell density was determined following the method of Rongshi Chen [38], fucoxanthin content and yield were measured according to Yiheng Song [15], and Chl a content was measured following the method of Hesheng Li [39].

### 4.8. Measurement of Chlorophyll Fluorescence Parameters Under the Optimal Inducer Combination

At 24 h, 48 h, and 72 h after treatment under the optimal inducer combination, NPQ, Y(II), Fv/Fm, and qP in *P. tricornutum* were measured according to the method described by Yiheng Song [15]. Three biological replicates were performed for each group.

### 4.9. Analysis of Fucoxanthin-Related Gene Expression Under the Optimal Inducer Combination

After 24 h of treatment under the optimal combination conditions, total RNA from *P. tricornutum* was extracted using the method of Yiheng Song [15] with the same reagent kit, and cDNA was synthesized from the total RNA.

Primers were designed using Oligo7 version 7.56 software for the photosynthesis-related genes (*crtiso*, *lcyb*, *pds*, *zds*, *zep*) and fucoxanthin biosynthetic pathway genes (*fcpb*, *psbA*, *rbcL*, *rbcS*). β-actin was used as the internal reference gene, and the reaction system and thermal cycling conditions were identical for target and reference genes. The RT-qPCR protocol followed Yiheng Song [15], and the melting curves were generated using the default settings of the instrument.

### 4.10. Data Processing and Statistical Analysis

Data were processed using Excel, while significance testing and correlation analysis were performed using SPSS 26.0. Fucoxanthin content and yield, Chl a content, chlorophyll fluorescence parameters, expression levels of photosynthetic key enzyme genes (*crtiso*, *lcyb*, *pds*, *zds*, *zep*) and fucoxanthin biosynthetic pathway key enzyme genes (*fcpb*, *psbA*, *rbcL*, *rbcS*) were compiled to form the raw dataset. The relative gene expression levels were calculated using the 2^−ΔΔCT^ method [40]. Principal component analysis was conducted using SPSS, and graphical plotting was performed with Origin 2024 software. Statistical significance was defined as *p* < 0.01 for extremely significant differences and *p* < 0.05 for significant differences.

## 5. Conclusions

This study is the first to elucidate the molecular mechanism by which Gly, light intensity and photoperiod synergistically regulate photosynthetic and metabolic gene expression to promote efficient fucoxanthin accumulation, providing a theoretical basis and optimization strategy for industrial-scale production. Future work may focus on constructing dynamic multi-factor regulatory models and applying metabolic engineering approaches to enhance the expression of key genes, thereby improving the economic feasibility and sustainability of large-scale fucoxanthin production.

## Figures and Tables

**Table 1:** Orthogonal experimental design and intuitive analysis of *P. tricornutum* (mean ± standard error, *n* = 3).

No.	Factors			Fucoxanthin Content (μg g^−1^)	Fucoxanthin Product (μg L^−1^)
	Gly	Light Intensity	Photoperiod		
1	1	1	1	0.83 ± 0.10 ± 0.07 ^c^	0.86 ± 0.07 ± 0.05 ^h^
2	2	2	1	0.93±0.01 ± 0.01 ^ab^	3.23 ± 0.07 ± 0.05 ^a^
3	3	3	1	0.85 ± 0.03 ± 0.02 ^bc^	2.72 ± 0.11 ± 0.08 ^b^
4	4	4	1	0.82 ± 0.04 ± 0.03 ^cde^	2.32 ± 0.06 ± 0.04 ^d^
5	2	1	2	0.97 ± 0.18 ± 0.13 ^a^	1.04 ± 0.06 ± 0.04 ^gh^
6	1	2	2	0.77 ± 0.03 ± 0.02 ^cde^	2.63 ± 0.08 ± 0.06 ^b^
7	4	3	2	0.80 ± 0.03 ± 0.02 ^cde^	2.68 ± 0.11 ± 0.08 ^b^
8	3	4	2	0.74 ± 0.04 ± 0.03 ^de^	2.63 ± 0.08 ± 0.06 ^b^
9	3	1	3	0.84 ± 0.03 ± 0.02 ^c^	1.28 ± 0.14 ± 0.10 ^fg^
10	4	2	3	0.80 ± 0.08 ± 0.06 ^cde^	2.61 ± 0.37 ± 0.26 ^bc^
11	1	3	3	0.73 ± 0.01 ± 0.01 ^e^	2.38 ± 0.14 ± 0.10 ^cd^
12	2	4	3	0.77 ± 0.04 ± 0.03 ^cde^	3.04 ± 0.13 ± 0.09 ^a^
13	4	1	4	0.85 ± 0.03 ± 0.02 ^bc^	1.46 ± 0.00 ± 0.00 ^f^
14	3	2	4	0.83 ± 0.01 ± 0.01 ^cd^	3.08 ± 0.08 ± 0.06 ^a^
15	2	3	4	0.79 ± 0.06 ± 0.04 ^cde^	3.15 ± 0.34 ± 0.24 ^a^
16	1	4	4	0.61 ± 0.08 ± 0.06 ^f^	1.73 ± 0.28 ± 0.20 ^e^
k_C_1	0.73	0.87	0.86	-	-
k_C_2	0.86	0.83	0.82	-	-
k_C_3	0.81	0.79	0.78	-	-
k_C_4	0.82	0.73	0.77	-	-
k_P_1	1.90	1.16	2.28	-	-
k_P_2	2.62	2.89	2.25	-	-
k_P_3	2.43	2.73	2.33	-	-
k_P_4	2.27	2.43	2.36	-	-
R_C_	0.13	0.14	0.09	-	-
R_P_	0.72	1.73	0.11	-	-

Note: k_C_1, k_C_2, k_C_3 and k_C_4 represent the average fucoxanthin content (μg g^−1^) for each experimental factor (glycine, light intensity, photoperiod) at four levels (1–4). These values are calculated by grouping data according to the same level of a single factor (e.g., all experiments with Gly at level 1) and computing their mean. Similarly, k_P_1 to k_P_4 denote the average fucoxanthin yield (μg L^−1^) for each factor-level combination. All mean values in this experiment are averages of biological replicates. R_C_ (content range) and R_P_ (yield range) quantify the relative influence of each factor by calculating the difference between the maximum and minimum values of k_C_ or k_P_. The letters a–h indicate the significance of differences. Identical letters signify no significant differences, while different letters indicate significant differences (*p* < 0.05).

**Table 2:** Variance analysis of the orthogonal experimental results of fucoxanthin content in *P. tricornutum*.

Source of Variation	Square Sum	Grees of Freedom	Mean Square	F	*p*	Significance
Gly	0.04	3	0.01	12.44	0.01	**
Light intensity	0.04	3	0.01	14.69	0.00	**
Photoperiod	0.02	3	0.01	7.02	0.02	*
Error	0.01	6	0.00			

Note: The square’s sum reflects the degree of variation in fucoxanthin content caused by each factor. The larger the square’s sum, the greater the contribution of the factor to the variation in fucoxanthin content. The mean square is used to measure the average degree of variation brought about by each factor. The F-value is used to determine whether a factor has a significant effect on fucoxanthin content. The larger the F-value, the more significant the effect of the factor. The F-value of light intensity is 14.69, which is greater than the F-value of Gly (12.44) and the F-value of photoperiod (7.02), indicating that among these factors, the light intensity factor has a relatively more significant effect on fucoxanthin content. The P-value represents the probability of rejecting the null hypothesis (i.e., the factor has no effect on fucoxanthin content) in a hypothesis test. When the *p*-value is less than a given significance level (usually 0.05), the factor is considered to have a significant effect on fucoxanthin content. In the table, the *p*-values of Gly, light intensity, and photoperiod are all less than 0.05, indicating that these three factors all have a significant effect on fucoxanthin content. “**” indicates extremely significant (*p*-value is much less than 0.05), and “*” indicates significant (*p*-value is slightly less than 0.05).

**Table 3:** Variance analysis of the orthogonal experimental results of fucoxanthin yield in *P. tricornutum*.

Source of Variation	Square Sum	Degrees of Freedom	Mean Square	F	*p*	Significance
Gly	1.11	3	0.37	3.76	0.08	**
Light intensity	7.39	3	2.46	24.98	0.00	**
Photoperiod	0.03	3	0.01	0.10	0.96	
Error	0.59	6	0.10			

Note: This table has a similar meaning to Table 2. The difference is that this table is related to the fucoxanthin yield, while Table 2 is related to the fucoxanthin content. “**” indicates extremely significant (*p*-value is much less than 0.01).

**Table 4:** The correlation and significance between the content and yield of fucoxanthin and various indicators.

Items		Indicators													
		Chla	F_v_/F_m_	Y(II)	qP	NPQ	*crtiso*	*lcyb*	*pds*	*zds*	*zep*	*fcpb*	*psbA*	*rbcL*	*rbcS*
C	Correlation coefficient	0.18	0.78	0.75	0.85	−0.90	0.28	0.15	0.02	0.10	0.15	0.23	0.64	0.33	0.93
	Sig. (both sides)	0.64	0.01	0.02	0.00	0.00	0.47	0.71	0.97	0.80	0.70	0.55	0.07	0.38	0.00
P	Correlation coefficient	0.98	0.35	0.71	−0.07	−0.30	0.91	0.95	0.95	0.94	0.94	0.95	0.74	0.80	0.12
	Sig. (both side)	0.00	0.36	0.03	0.86	0.43	0.00	0.00	0.00	0.00	0.00	0.00	0.02	0.01	0.75

Note: The correlation coefficient reflects the degree of linear correlation between fucoxanthin content or yield and various indicators. Its value ranges from −1 to 1. A positive value indicates a positive correlation, meaning that when one variable increases, the other variable also tends to increase; a negative value indicates a negative correlation, meaning that when one variable increases, the other variable tends to decrease. The two-tailed significance is used to determine whether the correlation coefficient is statistically significant. Usually, when this value is less than a given significance level (such as 0.05), the correlation represented by the correlation coefficient is considered significant; that is, it is not caused by random factors. For fucoxanthin content (C), the Sig. values of Fv/Fm, Y(II), qP, and NPQ are all less than 0.05, indicating that the correlations between these indicators and fucoxanthin content are significant; for fucoxanthin yield (P), except for a few indicators (such as the indicator corresponding to −0.07), the Sig. values of most indicators are less than 0.05, indicating that the correlations between these indicators and fucoxanthin yield are significant.

**Table 5:** Design table for the header of the L12(34) orthogonal test of *P. tricornutum*.

No.	Gly (g L^−1^)	Light Intensity (μmol Photons·m^−2^·s^−1^)	Photoperiod (h Light/h Dark)
1	0	18	12/12
2	0.5	36	16/8
3	1.0	54	20/4
4	2.0	72	24/0

## Data Availability

Data are contained within the article.

## References

[1] Zhu J.W., Ye Y.M., Zhou C.X., Zhang J.R. (2024). Isolation and Purification of Fucoxanthin from *Phaeodactylum tricornutum*. Chin. J. Mar. Drugs.

[2] Butler T., Kapoore R.V., Vaidyanathan S. (2020). *Phaeodactylum tricornutum*, A Diatom Cell Factory. Trends Biotechnol..

[3] Wang S., Wu S., Yang G., Pan K., Wang L., Hu Z. (2021). A Review on the Progress, Challenges and Prospects in Commercializing Microalgal Fucoxanthin. Biotechnol. Adv..

[4] Chen W., Zhang H., Liu Y. (2019). Anti-Inflammatory and Apoptotic Signaling Effect of Fucoxanthin on Benzo(A)pyrene-Induced Lung Cancer in Mice. J. Environ. Pathol. Toxicol. Oncol..

[5] Yang H.Y., Xing R.E., Liu S., Yu H.H., Li P.C. (2021). Role of Fucoxanthin towards Cadmium-Induced Renal Impairment with the Antioxidant and Anti-Lipid Peroxide Activities. Bioengineered.

[6] Koo S.Y., Hwang J.H., Yang S.H., Um J.I., Hong K.W., Kang K., Pan C.H., Hwang K.T., Kin S.M. (2019). Anti-Obesity Effect of Standardized Extract of Microalga *Phaeodactylum tricornutum* Containing Fucoxanthin. Mar. Drugs.

[7] Ahmed S.A., Mendonca P., Elhag R., Soliman K.F.A. (2022). Anticancer Effects of Fucoxanthin through Cell Cycle Arrest, Apoptosis Induction, Angiogenesis Inhibition, and Autophagy Modulation. Int. J. Mol. Sci..

[8] Liu Y., Zhi L.C., Wang H.R., Zhao L., Ren D.D., He Y.H., Wang Q.K. (2023). Inhibition of α-Glucosidase Activity and Hypoglycemic Effect by Fucoxanthin Extracted from Seaweed (*Sargassum horneri*). J. Dalian Ocean. Univ..

[9] Lee A.H., Shin H.Y., Park J.H., Koo S.Y., Kim S.M., Yang S.H. (2021). Fucoxanthin from Microalgae *Phaeodactylum tricornutum* Inhibits Pro-Inflammatory Cytokines by Regulating both NF-κB and NLRP3 Inflammasome Activation. Sci. Rep..

[10] Sarah M., Fodil M., Fleury F., Chénais B. (2020). Fucoxanthin, A Marine-Derived Carotenoid from Brown Seaweeds and Microalgae, A Promising Bioactive Compound for Cancer Therapy. Int. J. Mol. Sci..

[11] Kim S.M., Jung Y.J., Kwon O.N., Cha K.H., Um B.H., Chung D., Pan C.H. (2012). A Potential Commercial Source of Fucoxanthin Extracted from the Microalga *Phaeodactylum tricornutum*. Appl. Biochem. Biotechnol..

[12] Wang W.D., Yu L.J., Xu C.Z., Tomizaki T., Zhao S.H., Umena Y., Chen X.B., Qin X.C., Xin Y.Y., Suga M. (2019). Structural Basis for Blue-Green Light Harvesting and Energy Dissipation in Diatoms. Science.

[13] Xie Y., Chen Z.P., Ge S.J., Yang C., Qiu S. (2024). Effects of Light Intensity on Growth of Native Microalgal Cellular Components and Performance of Biodiesel. J. Nanjing Univ. Sci. Technol..

[14] Tong L.H., Wu X.Y., Huang L.F., Zeng J., Shi Y.H., Tang X.M. (2021). Correlation Analysis of Light Intensity and Growth, Photosynthetic Pigment, Color Value of *Betaphycus gelatinae*. S. China Fish. Sci..

[15] Song Y.H., Gong Y.F., Liu B.Y., Li L.Q., Wang H.Y. (2024). Effect of Different Photoperiods on Growth, Fucoxanthin Content, Photosynthesis and Related Gene Expression of *Isochrysis zhanjiangensis*. J. Nucl. Agric. Sci..

[16] Wang S., Verma S.K., Inamullah H.S., Laurenz T., Ullrich M.S., Nikolai K. (2018). Changes in the Fucoxanthin Production and Protein Profiles in *Cylindrotheca closterium* in Response to Blue Light-Emitting Diode Light. Microb. Cell Fact..

[17] Fathy W.A., AbdElgawad H., Essawy E.A., Tawfik E., Abdelhameed M.S., Hammouda O., Korany S.M., Elsayed K.N.M. (2023). Glycine Differentially Improved the Growth and Biochemical Composition of *Synechocystis* sp. PAK13 and *Chlorella variabilis* DT025. Front. Bioeng. Biotechnol..

[18] Li Y., Cui L., Lei J.M., Li Q., Zhang X. (2014). Effects of Different Concentrations of Organic Affixture on the Growth and Secondary Metabolites Contents in Adventitious Roots of *Tripterygium wilfordii*. Plant Sci. J..

[19] Zhao L.X. (2005). The Research of Biologic Characteristic of *Dunaliella Salina*. Master’s Thesis.

[20] Wu X., Gong Y.F., Li L.Q., Gao X.W., Lv J., Wang H.Y. (2024). Effects of Orthogonal Design Optimized Elicitor Combinations on fucoxanthin Content and Photosynthetic Physiology and Gene Expression of *Phaeodactylum Tricornutum*. Chin. Pharm. J..

[21] Guo W.X., Qiao F., Luo X.F., Zhou X.Z., Zhou J.J. (2024). Effects of Light on the Growth and Physiology of Three Strains of *Polygonati rhizoma* Seedlings. Chin. J. Trop. Agric..

[22] Zang W., Meng X.Q., Su X.H., Wang J.Y., Li L.H., Jia M. (2024). Effects of Light Regulation on the Synthesis of Secondary Metabolites in Medicinal Plants. J. Pharm. Pract. Serv..

[23] Fang H.L., Qi X.W., Li Y.M., Yu X., Xu D.B., Liang C.Y., Li W.L., Liu X. (2020). De Novo Transcriptomic Analysis of Light-Induced Flavonoid Pathway, Transcription Factors in the Flower Buds of *Lonicera japonica*. Trees.

[24] Cai Y.Y., Zhu Z.L., Jia Z.K., Ma L.Y., Sang Z.Y., Wu N.S., Xu M.Y., Luo Q.Q., Deng Z.W., Wang J.W. (2024). Effects of Photoperiod on Leaf Nutrition and Photosynthetic Physiology of *Magnolia wufengensis* “Jiaohong 1”. NWF Res..

[25] Dong W., Li M.M., Li Z.A., Li S.L., Zhu Y., Hong X., Wang Z.C. (2020). Transcriptome Analysis of the Molecular Mechanism of Chrysanthemum Flower Color Change Under Short-Day Photoperiods. Plant Physiol. Biochem..

[26] Ren H.M., Ren Y.M., Zhou Y.C., Jia S.J., Li W., Huang Y. (2022). Effects of Glycine Spraying Concentration on the Growth of Spinach. Liaoning Agric. Sci..

[27] Niu J.J., Fang S.M., Wang Q.Y., Liang X.L. (2024). Effects of Different Concentrations of Glycine Mixed in Soil on Growth Characteristics of Rice Seedlings. Chin. Rice..

[28] Qin J.L., Yu M.J., Li X., Xu N., Duan S.S. (2012). Effect of Nitrogen Sources on the Growth of *Phaeocystis globosa*. Ecol. Sci..

[29] Zhang Z.J., Wang P. (2011). Optimization of Culture Conditions of *Chlorella* sp.. J. Food Sci. Technol..

[30] Fu H.H., He Y.H., Yin Y.Y., Hu W., Yang Y., Yue Z.G. (2023). Optimum Conditions for Regulating Content Change of Secondary Metabolites of *Gentiana macrophylla* by Orthogonal Method. Guihaia.

[31] Wang M.L., Jiang Y.L. (2018). Effects of Manganese on the Growth and Fluorescence Induction Kinetics of *Conticribra weissflogii*. Environ. Sci..

[32] Gururani M.A., Venkatesh J., Tran L.S.P. (2015). Regulation of Photosynthesis During Abiotic Stress-Induced Photoinhibition. Mol. Plant..

[33] Wilson R.H., Hayer H.M. (2018). Complex Chaperone Dependence of Rubisco Biogenesis. Biochemistry.

[34] Mikami K., Hosokawa M. (2013). Biosynthetic pathway and health benefits of fucoxanthin, an algae-specific xanthophyll in brown seaweeds. Int. J. Mol. Sci..

[35] Liang M.H., Zhu J.H., Jiang J.G. (2018). Carotenoids biosynthesis and cleavage related genes from bacteria to plants. Crit. Rev. Food Sci. Nutr..

[36] Guan T.T. (2020). A Preliminary Study on the cDNA Cloning and Function of *Undaria Pinnatifida Suringar* Encoding Phytoene Desaturase. Master’s Thesis.

[37] Wei F.J., Gong Y.F., Zhang L., Chen R.S., Wang H.Y., Yang B.D. (2022). Effects of Rapamycin on the Content of Fucoxanthin in *Phaeodactylum tricornutum* and the Expression of Key Enzyme Genes. J. Biol..

[38] Chen R.S., Zhang L., Wei F.J., Yuan L.Y., Zhao P., Wang H.Y., Gong Y.F. (2021). Effects of Mn^2+^ on Neutral Lipid Content, C4 Pathway, and Related Gene Expression in *Phaeodactylum tricornutum*. SSBM.

[39] Li H.S., Sun Q., Zhao S.J. (2000). Principle and Technology of Plant Physiological and Biochemical Experiments.

[40] Livak K.J., Schmittgen T.D. (2001). Analysis of Relative Gene Expression Data Using Real-Time Quantitative PCR and the 2^−ΔΔCT^ Method. Methods.

